# Antiviral Activities of Asarones and Rhizomes of *Acorus gramineus* on Murine Norovirus

**DOI:** 10.3390/v14102228

**Published:** 2022-10-10

**Authors:** Hyojin Kim, Jin Young Maeng, Dan Bi Lee, Kyung Hyun Kim, Mi Sook Chung

**Affiliations:** 1 Department of Food and Nutrition, Duksung Women’s University, Seoul 01369, Korea; 2 Department of Biotechnology and Bioinformatics, Korea University, Sejong 30019, Korea

**Keywords:** norovirus, antiviral, P domain, *Acorus gramineus* rhizome, asarone, simulated digestive condition

## Abstract

Noroviruses (NVs) are a major cause of foodborne diseases worldwide. The rhizomes of *Acorus gramineus* (AGR) have been used as a traditional medicinal plant and a food additive. In this study, AGR and its bioactive components—α-asarone and β-asarone—showed significant antiviral activities against murine NV (MNV) with pre-treatment, with more than two log reductions in viral plaques. They also demonstrated strong inhibition on binding to A- and O-type saliva by the recombinant P domain derived from human NV (HuNV) GII.4. Both α- and β-asarones also inhibited the binding of the P domain to the receptor at 0.125–1 mM in a concentration-dependent manner and induced a marked reduction in T_m_, suggesting that they may reduce structural stability and block receptor binding by the P domain. In simulated digestive conditions, the AGR extract, α-asarone, or β-asarone further showed a significant reduction of MNV plaques by 1.5–2.8 logs. The asarones show a potential for development as a scaffold for anti-NV agents.

## 1. Introduction

Noroviruses (NVs) are a major cause of infectious foodborne diseases worldwide. The global burden associated with NV-mediated foodborne diseases is estimated at approximately 65 billion dollars in annual medical and societal costs [1]. In the United States, the annual economic burden of NV reaches 10.6 billion dollars, including direct health costs and productivity loss [2]. NVs belonging to the *Caliciviridae* family can be classified into 10 genogroups (GI–GX) and 48 genotypes [3]. Among the genogroups, GI, GII, GIV, GVIII, and GIX are known to infect humans, and the GII.4 genotype accounts for the majority of outbreaks worldwide [3,4,5]. Human norovirus (HuNV) initiates infection by binding to a receptor, histo-blood group antigen (HBGA), using the protruding (P) domain of the capsid protein VP1 [6]. VP1 has shell (S) and P domains that contain the receptor binding site and determinants of antigenicity [7,8]. HBGA is complex glycan on the surface of mucosal epithelial cells and red blood cells and is present in milk, saliva, and the intestine [9]. HuNV was reported to be cultured in B cells and human intestinal enteroids [10,11]. Nevertheless, the high cost and availability of these HuNV systems have been barriers to the widespread use of improved culture methods. Murine norovirus (MNV) has been used as the predominant surrogate for HuNV due to its similar genomic organization and robust propagation in cell culture systems [12].

*Acorus gramineus* (Araceae) is an aquatic perennial plant which is distributed in China, Korea, and Japan. The rhizome of *A*. *gramineus* (AGR) has been widely used as traditional medicine to control cognitive disorders, sedation, and diarrhea [13,14,15]. AGR is rich in phytochemicals, including α-asarone, β-asarone, and alkaloids [14,16,17,18]. Asarones belong to the phenylpropanoid class in plants, and α and β isomers differ in the configuration of the propenyl group attached to their benzene ring. β-Asarone, the major component in AGR, shows improvement of cognitive function [19] and anti-allergic [20] and antifungal activity [16]—whereas α-asarone, a minor component in the AGR, shows neuroprotective activity [17,18].

In this study, we investigated the inhibitory activity of AGR and its bioactive components—α-asarone and β-asarone—against MNV and HuNV. In addition, the antiviral activities of AGR, α-asarone, and β-asarone under simulated in vitro digestive conditions are described.

## 2. Materials and Methods

### 2.1. Preparation of Cells, MNV, and AGR Extract

RAW 264.7 cells (mouse leukemic macrophage cell line; RAW) were purchased from ATCC and grown in DMEM (Gibco BRL, Karlsruhe, Germany) supplemented with 1% penicillin–streptomycin (PS, Gibco BRL) and 10% fetal bovine serum (FBS, Sigma-Aldrich, St. Louis, MO, USA) at 37 °C and 5% CO_2_. MNV-1 was kindly provided by Dr. Herbert Virgin (Vir Biotechnology, San Francisco, CA, USA). MNV was replicated and purified as previously reported [21]. The purified MNV suspension was stored as aliquots at −80 °C until its use.

The dried rhizome of *A. gramineus* (AGR; voucher no. DSNPL0020) was purchased from Booguk Co. (Seoul, Korea). AGR powder <1 mm in size was extracted with 70% ethanol in an ultrasonic bath at a frequency of 42 kHz (Hwashin Instrument Co., Ltd. Seoul, Korea) and 20 °C for 1 h. The extract was centrifuged at 1610× *g* at 4 °C for 30 min and the supernatant was lyophilized (IlShin BIOBASE, Seoul, Korea). The percentage yield of the AGR extract was calculated as the dry weight obtained after lyophilization and was divided by the sample weight and multiplied by 100. The AGR extract yield (%) was 12.9%.

### 2.2. Cell Viability Assay

The AGR extract was serially diluted 10-fold using DMEM. α-Asarone (>99%, Aktin Chemicals, Inc., Chengdu, China) or β-asarone (>98%, Aktin Chemicals, Inc., Chengdu, China) was dissolved in dimethyl sulfoxide (DMSO; 10 mM in 2.2% DMSO) and then diluted in DMEM to a working concentration. 10 µL of AGR extract, α-asarone, or β-asarone, and 90 µL of DMEM supplemented with 1% PS and 10% FBS were added to RAW 264.7 cells, which were grown to >90% confluence in 96-well plates. For the cell control, 10 µL of DMEM containing 2.2% DMSO was used instead of the sample. The plate was incubated at 5% CO_2_ and 37 °C for 24 h. A 3-(4,5-dimethylthiazol-2-yl)-2,5-diphenyltetrazolium bromide (MTT, Sigma-Aldrich) solution was added to each well of the plate. After incubation for 1 h, dimethyl sulfoxide was added, and the absorbance was measured at 570 nm using a microplate reader (SpectraMax M2, Molecular Devices, San Jose, CA, USA). Cell viability (%) was calculated as follows: (Abs_sample_/Abs_control_) × 100.

Lactate dehydrogenase (LDH) was measured for cell death assessment using the EZ-LDH cytotoxicity assay kit (DoGenBio Co., Seoul, Korea). A total of 100 µL of RAW 264.7 cells were seeded at 2 × 10^6^ cells/mL in a 96-well plate and cultured for 24 h before being incubated with 50 µL of AGR (0.125–1 mg/mL), α-asarone (0.125–1 mM), or β-asarone (0.125–1 mM) for 24 h. For the blank (RAW 26.7 cell control), 50 µL of DMEM containing 2.2% DMSO was used instead of the sample. After incubation, incubated cells were centrifuged at 600× *g* for 5 min. Then, 10 µL of the supernatant was transferred into a new 96-well plate. Positive control cells were lysed using 10 µL of the lysis solution provided in the assay kit. LDH released into the extracellular medium was quantified by the assay kit according to the manufacturer’s instructions. Fresh culture medium was used as a blank. The absorbance was measured at 450 nm using a microplate reader (SpectraMax M2, Molecular Devices). LDH (%) = (Abs_sample_−Abs_blank_)/(Abs_positive control_−Abs_blank_) × 100. The mean value of LDH for the positive control (lysis solution; 0.1% triton X-100) was considered to be 100% [22].

### 2.3. Plaque Assay

The antiviral effects of the AGR extract, α-asarone, and β-asarone were analyzed by plaque assays [23]. For the pre-treatment of MNV, the AGR extract (0.125–1 mg/mL), α-asarone (0.125–1 mM), β-asarone (0.125–1 mM), or combination of α- and β-asarone (1:1 ratio) were incubated with the MNV suspension (7–8 log PFU/mL) at a 1:1 ratio at room temperature for 3 h. The incubated suspension was 10-fold serially diluted with DMEM and then added to confluent RAW 264.7 cell monolayers in a 24-well plate at 37 °C and 5% CO_2_. After 1 h incubation, the added suspension was removed, and 1 mL of DMEM supplemented with 0.5% PS, 5% FBS, and 1% agarose was added to each well. The plate was incubated at 37 °C and 5% CO_2_ for 48 h, and the cells were fixed with 4% formaldehyde for 1 h. The agarose was removed, and the cells were stained with 0.5% crystal violet solution. For the co-treatment, confluent cell monolayers were infected with 200 μL of viral suspension (2–3 log PFU/mL) and simultaneously mixed with the AGR extract, α-asarone, or β-asarone. The rest of the experimental procedure was the same as in the pre-treatment of MNV. The number of plaques was counted in each well. The 2-Thiouridine (2TU) and DMEM-containing DMSO (a final concentration of 0.22%) were used as positive and untreated controls, respectively.

### 2.4. Expression and Purification of HuNV GII.4 P domain

The DNA fragments encoding the P domain of HuNV GII.4 (Hu/GII.4/Hiroshima/55/2005/JPN; GenBank accession number BAI49908.1) were synthesized by Macrogen (Seoul, Korea). The GII.4 P domain was expressed and purified as previously described, with minor modifications [24]. The gene was cloned into the pET14b vector (Novagen, Madison, WI, USA) and transformed into *Escherichia coli* BL21 (DE3) competent cells (Novagen). The cells were centrifuged at 3500× *g* for 15 min and sonicated, and then centrifuged at 12,000× *g* for 20 min. The supernatant containing the GII.4 P domain was purified by Ni-NTA affinity chromatography (Qiagen, Hilden, Germany) and Superdex 200 chromatography (GE Healthcare, Uppsala, Sweden).

### 2.5. Enzyme-Linked Immunosorbent Assay (ELISA)

The ELISA assay was performed as described previously [24]. A- and O-type of saliva were obtained from volunteers at Duksung Women’s University. ELISA plates were coated with 100 µL of saliva, which was diluted at 1:100 with PBS. The plates were blocked with 5% non-fat dried milk at 37 °C for 1 h and washed three times with PBS. The GII.4 P domain was incubated with the AGR extract (0.125–1 mg/mL), α-asarone (0.125–1 mM), or β-asarone (0.125–1 mM) at 4 °C overnight. After incubation, the P domain was added to the coated plates, which were incubated at 37 °C for 1 h, and washed three times with PBS. Biotin-conjugated anti-GII.4 P domain antibodies (R-Biopharm AG, Darmstadt, Germany) were incubated at 37 °C for 1 h and washed three times with PBS. Streptavidin polyperoxidase-conjugated antibody (R-Biopharm AG) was then added to the plates and washed four times with PBS. o-Phenylenediamine and hydrogen peroxide were incubated at room temperature for 15 min, and the reaction was stopped with 2 N hydrochloric acid. The absorbance was measured at 450 nm using a microplate reader (SpectraMax M2). The absorbance of the untreated P domain was set as 100% saliva binding. The percentage of inhibition was calculated as follows: [1 − (Abs_treated P domain_/Abs_untreated P domain_)] × 100. A commercial fucoidan from *Undaria pinnatifida* (≥95% purity, Sigma-Aldrich) was used as a control.

### 2.6. Differential Scanning Fluorimetry (DSF)

DSF analysis was performed as described previously [25]. The HuNV GII.4 P domain (0.2 mg/mL) was incubated with α-asarone (2 mM) or β-asarone (2 mM) at 4 °C overnight. The incubated solution was mixed with 0.5 µL SYPRO Orange and incubated for 30 s at 25 °C. The temperature was increased for 80 min at the rate of 0.5 °C/30 s, and the relative fluorescence unit was measured using excitation/emission at 492/610 nm (Mx3005P DSF, Stratagene, CA, USA). The melting temperature (T_m_) was determined by calculating the maxima of the first derivative of relative fluorescence units/temperature using MxPro QPCR software.

### 2.7. Glutathione (GSH) Assay

RAW cells were seeded in a 24-well plate at a density of 0.5 × 10^6^ cells per well and grown to ~90% confluency [26]. The experimental groups were (1) cell as a control, (2) cell + MNV infection, and (3) cell + MNV infection + co-treated with AGR extract at 0.125–1 mg/mL. The treated cells were incubated for 3 h and centrifuged at 1610× *g* for 30 min. The supernatant (cell lysate) was collected and mixed with GSH reductase (Sigma-Aldrich) and 5,5′-dithio-bis(2-nitrobenzoic acid; Sigma-Aldrich). Next, β-nicotinamide adenine dinucleotide 2′-phosphate (NADPH) tetrasodium salt hydrate reduced form was added, and then the absorbance at 412 nm was measured. The GSH levels were expressed as % of nM/10^6^ cells.

### 2.8. Ferric Reducing Antioxidant Power Assay

Ferric reducing antioxidant power (FRAP) reagent was freshly prepared by mixing 0.3 M acetate buffer at pH 3.6, 20 mM FeCl_3_.6H_2_O, and 10 mM tripyridyltriazine solution in 40 mM HCl [27]. Then, 50 µL of AGR extract was mixed with 150 µL of pre-warmed FRAP reagent, and the absorbance was measured at 593 nm. The calibration curve of Fe(II) at concentrations of 15.6–125 µM was prepared (R^2^ = 0.999). The FRAP values were reported as µM of ferrous iron equivalent.

### 2.9. DPPH Radical Scavenging Activity Assay

A total of 100 µL of the AGR extract was mixed with 100 µL of 0.3 mM 2,2-diphenyl-1-picrylhydrazyl (DPPH; Sigma-Aldrich) [28]. The mixture was incubated for 30 min and the absorbance at 515 nm was determined. The calibration curve of ascorbic acid (1–50 µg/mL) was prepared (R^2^ = 0.999). The % of scavenging activity was calculated as follows: [(Abs_control_ − Abs_sample_)/Abs_control_] × 100.

### 2.10. Antiviral Effects of AGR Extract, α-Asarone, and β-Asarone under Simulated Digestive Conditions

Antiviral effects of the AGR extract, α-asarone, or β-asarone in simulated digestive conditions were analyzed. Simulated saliva fluid (SSF) for the mouth, simulated gastric fluid (SGF) for the stomach, and simulated intestinal fluid (SIF) for the small intestine were prepared: the SSF contained 150 unit/mL of salivary α-amylase at pH 7, the SGF contained 4000 unit/mL of porcine pepsin at pH 3, and the SIF had 200 unit/mL of pancreatin and 20 mM of bile at pH 7 [29].

The experiments were performed in dark conditions, and all incubations were carried out in a shaking water bath at 90 stroke/min and 37 °C [30]. First, the SSF was mixed with MNV suspension (a final titer of 6 log PFU/mL) and the AGR extract (a final concentration of 1 mg/mL), α-asarone (1 mM), or β-asarone (1 mM), which was incubated for 2 min. The mixture of 2 mL was then mixed with SGF (1:1, *v/v*) and re-incubated for 2 h. The re-incubated SGF mixture was mixed with GIF (1:1, *v/v*) and further incubated for 2 h and 10-fold serially diluted in DMEM. The diluted solution was inoculated onto confluent RAW 264.7 cell monolayers at 5% CO_2_ and 37 °C for 1 h. The remaining procedure was as described for the plaque assay method.

### 2.11. HPLC Analysis of AGR Extract

The contents of α-asarone or β-asarone in the AGR were analyzed using a UHPLC system (Thermo Scientific™ Dionex™ UltiMate™ 3000, MA, USA) with an ACE 5 C18-300 column (150 × 4.6 mm; Apex Scientific Limited, Maynooth, Ireland). The mobile phase was distilled water (A) and 100% methanol (B). The gradient elution was 0–3 min, 50% B; 3–12 min, 50–60% B; 12–24 min, 60% B; 24–24.6 min, 60–100% B; 24.6–30 min 100% B. The flow rate was 1 mL/min and the injection volume was 100 µL. The detection wavelength was 211 nm [31].

The contents of α-asarone and β-asarone in the AGR extract were calculated using the calibration curve. The stock solution of 200 mM of α-asarone or β-asarone was prepared and diluted serially and injected into HPLC for the calibration curve. The data for the compound concentration versus the peak area were analyzed with linear regression using Excel software (R^2^ = 0.994 for α-asarone and R^2^ = 0.997 for β-asarone).

### 2.12. Data Analysis

In this study, all assays were performed in triplicate. The statistical analyses were carried out using IBM SPSS Statistics (version 26, IBM Corp, New York, NY, USA). ANOVA with Tukey’s test was performed for multiple comparisons. *p* < 0.05 was considered statistically significant, with mean ± SD.

## 3. Results

### 3.1. AGR Extract, α-Asarone, and β-Asarone Show Antiviral Effects against MNV

**Figure 1:**
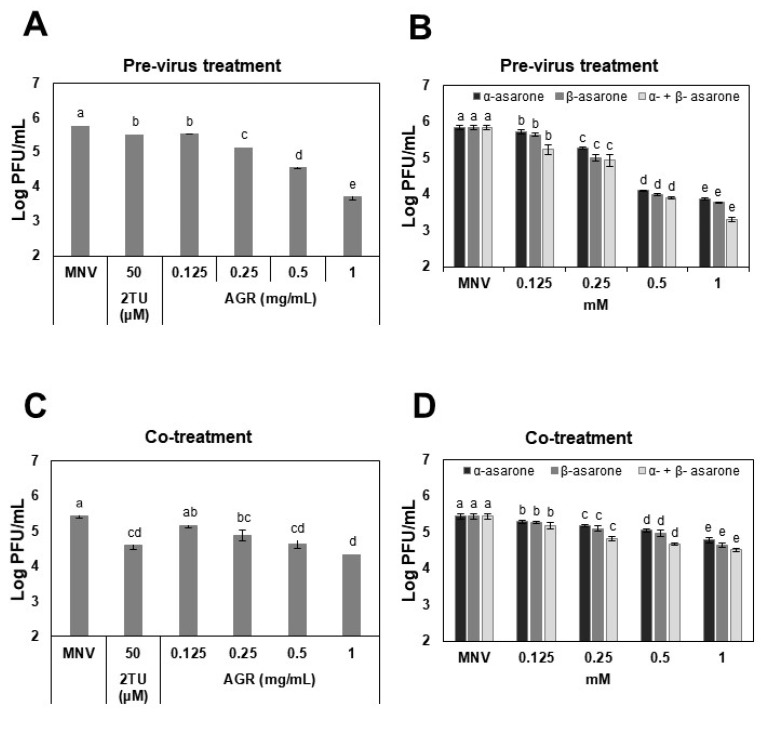
Antiviral effects of the AGR extract, α-asarone, and β-asarone against MNV. For the pre-treatment of MNV, (**A**) AGR extract (0.125–1 mg/mL), (**B**) α-asarone (0.125–1 mM), β-asarone (0.125–1 mM), or a combination of α- and β-asarone (1:1 ratio, 0.125–1 mM) was incubated with the MNV suspension (7–8 log PFU/mL) at a 1:1 volume ratio at room temperature for 3 h. The incubated suspension was added to confluent RAW 264.7 cells. For the co-treatment, confluent cell monolayers were infected with 200 μL viral suspension (2–3 log PFU/mL) and simultaneously mixed with (**C**) AGR extract (0.125–1 mg/mL), (**D**) α-asarone (0.125–1 mM), β-asarone (0.125–1 mM), or a combination of α- and β-asarone (1:1 ratio, 0.125–1 mM). The 2-thiouridin (2TU) and PBS were the positive and untreated controls, respectively. Different letters designate significant differences (*p* < 0.05).

To assess the antiviral activity of the AGR extract, α-asarone, and β-asarone, plaque assays were performed. In the pre-treatment of MNV, the extract showed dose-dependent inhibition at 0.125–1 mg/mL, achieving a 2.2 log reduction at 1 mg/mL (*p* < 0.05; Figure 1A). For comparison, 2TU used as a positive control yielded only a 0.7 log reduction at 50 µM. α-Asarone and β-asarone also showed antiviral effects in a dose-dependent manner at 0.125–1 mM (Figure 1B), reaching 2.1 and 2.0 log reductions of MNV titers, respectively. There were no significant differences in antiviral effects between α- and β-asarone alone or combined on MNV (Figure 1B). The contents of α-asarone and β-asarone in the AGR extract were measured using UHPLC, to show that they were 61.3 ± 1.9 and 857.2 ± 31.9 µg/mL, respectively (Figure S1).

In the co-treatment, the AGR extract at 0.125–1 mg/mL induced a 0.3–1.1 log reduction in MNV titers (Figure 1C), while 2TU at 50 µM showed a 0.5 log reduction. α-Asarone and β-asarones at 0.125–1 mM showed 0.2–0.8 log reductions in the viral titers (Figure 1D). There was no combination effect of α-asarone and β-asarone on MNV (Figure 1D). These results demonstrate that the extract, α-asarone, and β-asarone had significant antiviral effects on MNV and the pre-treatment with the virus consistently showed a higher antiviral effect than the co-treatment. In addition, the extract, α-asarone, and β-asarone were found to induce no cytotoxic effect at concentrations used in this study. RAW cells showed >94% viability in the presence of the extract at 0.125–1 mg/mL or α-asarone or β-asarone at 0.125–1 mM upon 24 h incubation using an MTT assay (Figure S2). In addition, the LDH activity was found to be below 8% using an LDH assay (Figure S2).

### 3.2. AGR Extract, α-Asarone, and β-Asarone Inhibit the Binding of HuNV P Domain to a Receptor

**Figure 2:**
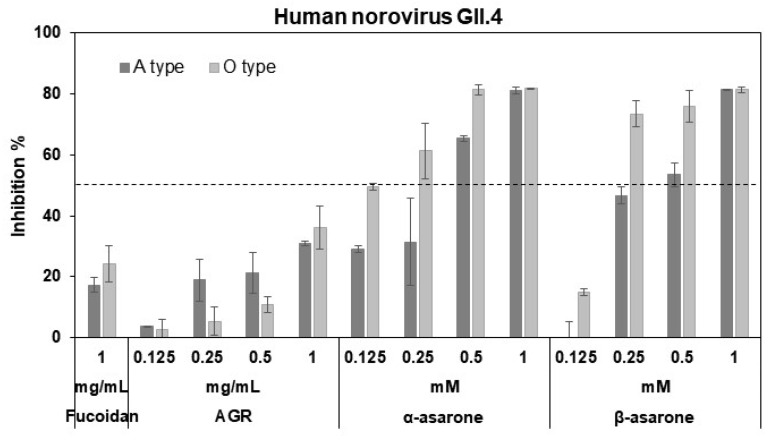
Inhibition of binding of the HuNV GII.4 P domain to saliva by AGR extract, α-asarone, and β-asarone. Inhibition was analyzed by ELISA assay. A positive control was a commercial fucoidan and the dashed line designates 50% inhibition.

As the pre-treatment of MNV provided higher antiviral effects than the co-treatment, ELISA assays using A- or O-type saliva were employed to determine whether inhibition by the AGR extract occurs at the receptor binding of the HuNV P domain. Binding of the GII.4 P domain to saliva was significantly inhibited by the extract, α-asarone, and β-asarone (Figure 2). α-Asarone at 0.5 mM achieved 65–81% inhibition of binding of the GII.4 P domain to A- and O-type saliva, while the commercial fucoidan—as a positive control—revealed 17–24% inhibition at 1 mg/mL. The extract, α-asarone, and β-asarone thus inhibit the binding of the GII.4 P domains to receptors. The α-asarone was significantly more effective at low concentrations such as 0.125 mM compared to the other isomer.

**Figure 3:**
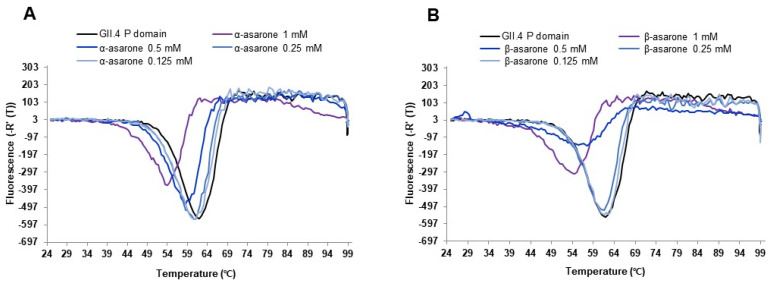
Differential scanning fluorimetry (DSF) profile of the effect of α-asarone and β-asarone on the HuNV GII.4 P domain stability. The effect of (**A**) α-asarone or (**B**) β-asarone on P domain stability was evaluated using DSF assay.

To further examine the effect of the asarone isomers on the HuNV GII.4 P domain, we examined the melting temperature T_m_ of the P domain in the absence and presence of α-asarone or β-asarone using the DSF assay. The T_m_ of the P domain was significantly decreased (−4–−8 °C) by α-asarone or β-asarone at 0.5–1 mM (Figure 3A,B). The results suggest that, instead of specific binding of α-asarone or β-asarone to the P domain, the isomers may reduce the overall stability of the HuNV GII.4 P domain as the concentrations increase.

### 3.3. Antioxidant Effects of AGR Extract in MNV-Infected Cells

**Figure 4:**
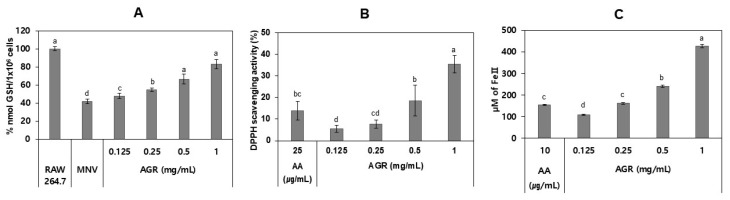
Antioxidant activities of AGR extract. (**A**) GHS levels in MNV-infected cells, (**B**) DPPH radical scavenging activity, and (**C**) FRAP assay. Ascorbic acid (AA) was used as a positive control in DPPH scavenging and FRAP assays. Different letters designate significant differences (*p* < 0.05).

To determine the antioxidant activity of the AGR extract in infected cells, we examined GSH levels, DPPH scavenging, and ferric ion-reducing activities. The MNV-infected cells treated with the extract exhibited significantly increased GSH levels at 0.125–1 mg/mL, with a maximum enhancement of 41.1%, compared to those in the MNV-infected cells (*p* < 0.05; Figure 4A). The extract also demonstrated significant DPPH scavenging and ferric ion-reducing activities in a dose-dependent manner (*p* < 0.05) with an increase of 34.6% in scavenging activity at 1 mg/mL (Figure 4B) and ferrous equivalent values of 161.3 µM at 0.25 mg/mL (Figure 4C). As a control, ascorbic acid showed a DPPH scavenging activity of 13.9% at 25 µg/mL and ferrous equivalent values of 154.6 µM at 10 µg/mL.

### 3.4. AGR Extract, α-Asarone, and β-Asarone Show Antiviral Effects under Simulated Digestive Conditions

**Figure 5:**
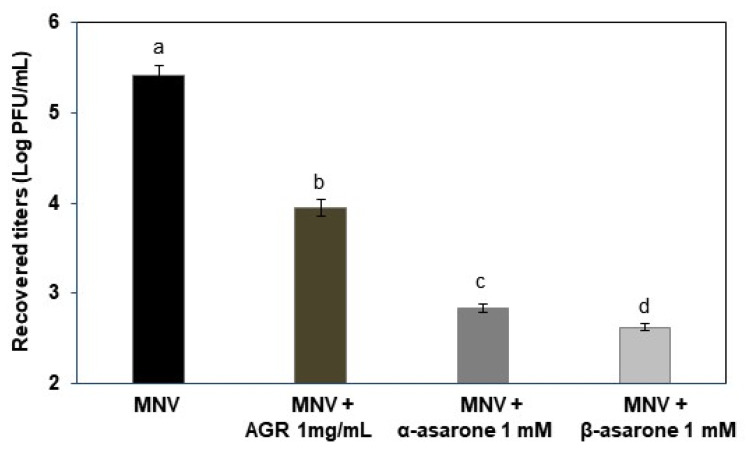
Recovered titers of MNV after treatment with AGR extract, α-asarone, or β-asarone under simulated digestive conditions. MNV titers were evaluated using plaque assays. Different letters designate significant differences (*p* < 0.05).

To investigate the antiviral activity of the AGR extract, α-asarone, or β-asarone in the digestive system, we used simulated digestive conditions in the presence of MNV. The extract at 1 mg/mL and α- or β-asarone at 1 mM significantly reduced MNV titers by 1.5, 2.6, and 2.8 logs, respectively (*p* < 0.05), compared to the control (Figure 5).

## 4. Discussion

NV is responsible for almost a fifth of all-cause acute gastroenteritis worldwide, causing approximately 698.8 million cases of disease and 218,800 deaths yearly in all age groups [1,32]. However, there are neither prophylactic vaccines for sufficient protection nor antiviral drugs to treat the virus effectively yet. There are many plant-derived natural products for controlling NVs, including black raspberry seeds, *Laminaria japonica* fucoidan, aged-green tea, and blueberry proanthocyanidin, [23,24,33,34]. In this study, the AGR extract, α-asarone, and β-asarone showed a significant antiviral effect on MNV. Their pre-treatment with MNV showed stronger antiviral effects (>2 log reduction) than the co-treatment at a concentration of 1 mg/mL or 1 mM, respectively. In addition, the extract showed significant inhibition against the HuNV GII.4 P domain binding to saliva, where HBGA is present to serve for in vitro assays of HuNV–HBGA interactions [35]. The P domain that forms dimers binds to HBGAs, which is sufficient for strain specificity and with the same patterns as the intact viral capsid [36]. While a positive control fucoidan at 1 mg/mL showed ~24% inhibition, α-asarone and β-asarone at 1 mM inhibited ~81% of the GII.4 P domain binding. Notably, α-asarone is more potent in binding inhibition than β-asarone at low concentrations. The results suggest that the α-asarone can effectively inhibit HuNV GII.4 entry into host cells, possibly blocking the attachment of the viral capsid to the glycan receptor. The HuNV GI, GII, GIV, GVIII, and GIX genotypes cause infection in humans, of which GII.4 has been the predominant genotype in NV outbreaks during the last 40 years [3,37]—which is particularly associated with more severe symptoms compared to other genotypes in children [38].

Moreover, asarones decrease the T_m_ by almost 10 °C using DSF, which is widely used for measuring protein stability [39]. The considerable reduction in the T_m_ strongly suggests that the presence of asarones leads to decreased stability of the P domain. Antigenic variation of the P domain of the GII.4 capsid protein evades previously elicited antibodies [33], and it is likely that asarones inhibit the binding of the P domain to the receptor by reducing its structural stability and induce the loss of receptor binding by the P domain. AGR is an agricultural product used for the ingredients of food products such as tea beverages [40]. Asarones are a phenylpropanoid found in *Acorus* and *Asarum* [41]. Two isomers—α- and β-asarone—have been reported to have biological effects such as antifungal, neuroprotective, and anti-allergic activities [16,17,19,20,42]. However, β-asarone was reported to induce toxicity, including carcinogenicity, and is officially regulated in the European Union [41,43]. In this study, α-asarone and β-asarone in the AGR extract showed high antiviral activity. Therefore, unlike β-asarone, α-asarone has no toxicity issues, providing a potential for its development as a scaffold for antiviral agents against emerging novel variants of GII.4—including other genotypes.

Virus infection induces redox imbalance by decreasing GSH levels and enhancing ROS levels [44,45,46]. In this study, the AGR extract increased GSH in the MNV-infected cells (*p* < 0.05). In addition, the extract showed ferric ion-reducing and DPPH scavenging activities. It was previously reported that the hop extract and *Magnolia officinalis* extract increases GSH content in virus-infected cells, which inhibits virus replication [45,46]. In this context, it is likely that the AGR extract also ameliorates ROS damage by increasing GSH levels in MNV-infected cells, inhibiting MNV replication.

Many natural products with promising antiviral activities in vitro display reduced activity against NV under gastrointestinal conditions [33,34]. Mori Cortex Radicis, aged-green tea extract, and blueberry proanthocyanidins also reduce MNV titers under simulated conditions [21,33,34]. It has been reported that MNV capsid proteins are sensitive to the presence of bile, Mg^2+^, and Ca^2+^, and that at low pH—at which they are induced to contract between the P and the S domains—they enhance receptor binding [47]. However, even in simulated human digestive systems, including the low pH, digestive enzymes, and bile salts, the AGR extract, α-asarone, or β-asarone achieve a significant MNV reduction by 1.5–2.8 logs. These results suggest that they exhibit remarkable antiviral activity under the conditions encountered on passing through the gastrointestinal tract.

In conclusion, we demonstrate that AGR and its bioactive components—α-asarone and β-asarone—were shown to have significant in vitro inhibitory effects against MNV, and further showed significantly reduced MNV titers under simulated digestive conditions. The extract also showed strong inhibition of HuNV GII.4 P domain binding to saliva. P domain binding was significantly inhibited by the minor and major components of the AGR extract, α-asarone or β-asarone, respectively. These compounds induced a marked decrease in the T_m_, which suggests that the compounds reduced P domain stability markedly. α-asarone was without toxicity issues compared to β-asarone, which affected the overall stability of P domains at low concentrations. Taken together, this study provides evidence that α-asarone or the AGR extract could be developed as antiviral materials for controlling NV foodborne diseases.

## Data Availability

Not applicable.

## Supplementary Materials

The following are available online at https://www.mdpi.com/article/10.3390/v14102228/s1, Figure S1: HPLC chromatograms of the AGR extract only (black) and the extract with (A) α-asarone or (B) β-asarone as standards (blue). The AGR extract was used at 0.5 mg/mL, while the extract was used at 0.25 mg/mL with α-asarone or β-asarone at 0.25 mM. β-Asarone is a major component in the extract, Figure S2: Cytotoxicity of AGR extract, α-asarone, and β-asarone using (A, B) the MTT and (C, D) LDH assays. RAW cells were incubated with the AGR, α-asarone, β-asarone, or combination of α- and β-asarone for 24 h, respectively. The mean value of LDH of the positive control (lysis solution; 0.1% triton X-100) was considered as 100%.
