# Supplementary material for: Antiviral Activities of Asarones and Rhizomes of Acorus gramineus on Murine Norovirus
Source: Viruses. 2022 Oct 10;14(10):2228. doi: 10.3390/v14102228 (PMC9611275; doi:10.3390/v14102228)
Supplement: Supplementary file 1 [file viruses-14-02228-s001.zip › viruses-1902348-supplementary.pdf]

viruses

MDPI

Article

Supplementary Materials: Antiviral activities of α-asarone and rhizomes of *Acorus gramineus* on norovirus

Hyojin Kim[1], Dan Bi Lee[2], Jin Young Maeng[1], Kyung Hyun Kim[2,*], and Mi Sook Chung[1,*]

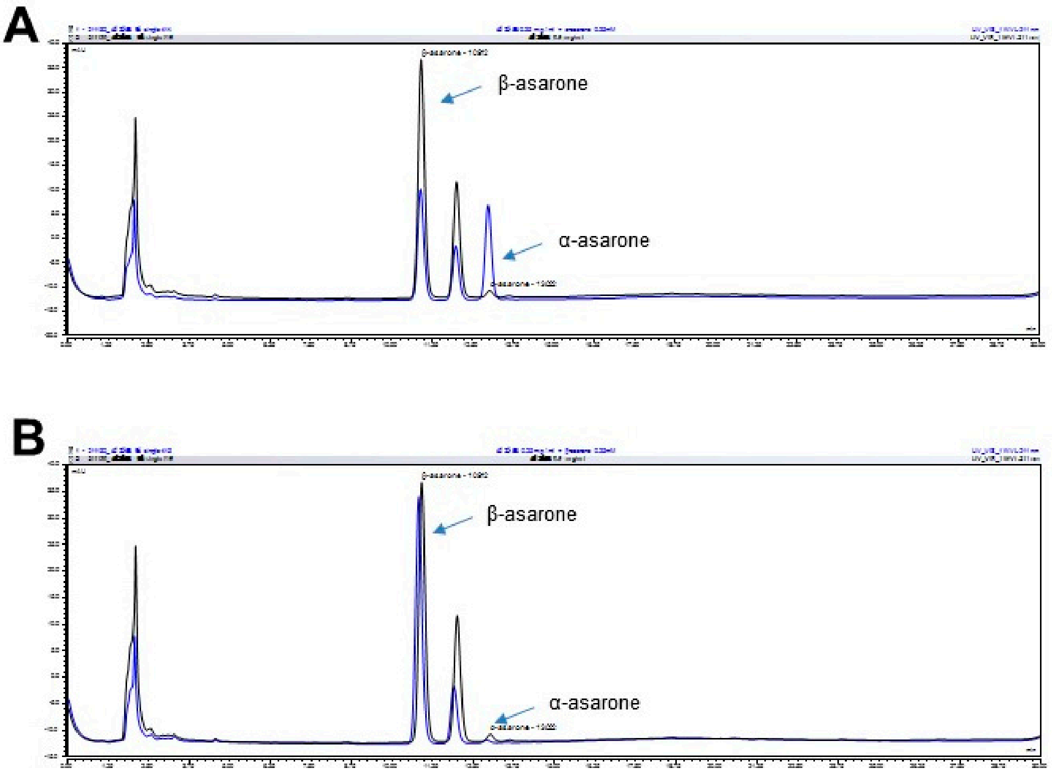

Figure S1. HPLC chromatograms of the AGR extract only (black) and the extract with (A) α-asarone or (B) β-asarone as standards (blue). The AGR extract was used at 0.5 mg/mL, while the extract was used at 0.25 mg/mL with α-asarone or β-asarone at 0.25 mM. β-Asarone is a major component in the extract.

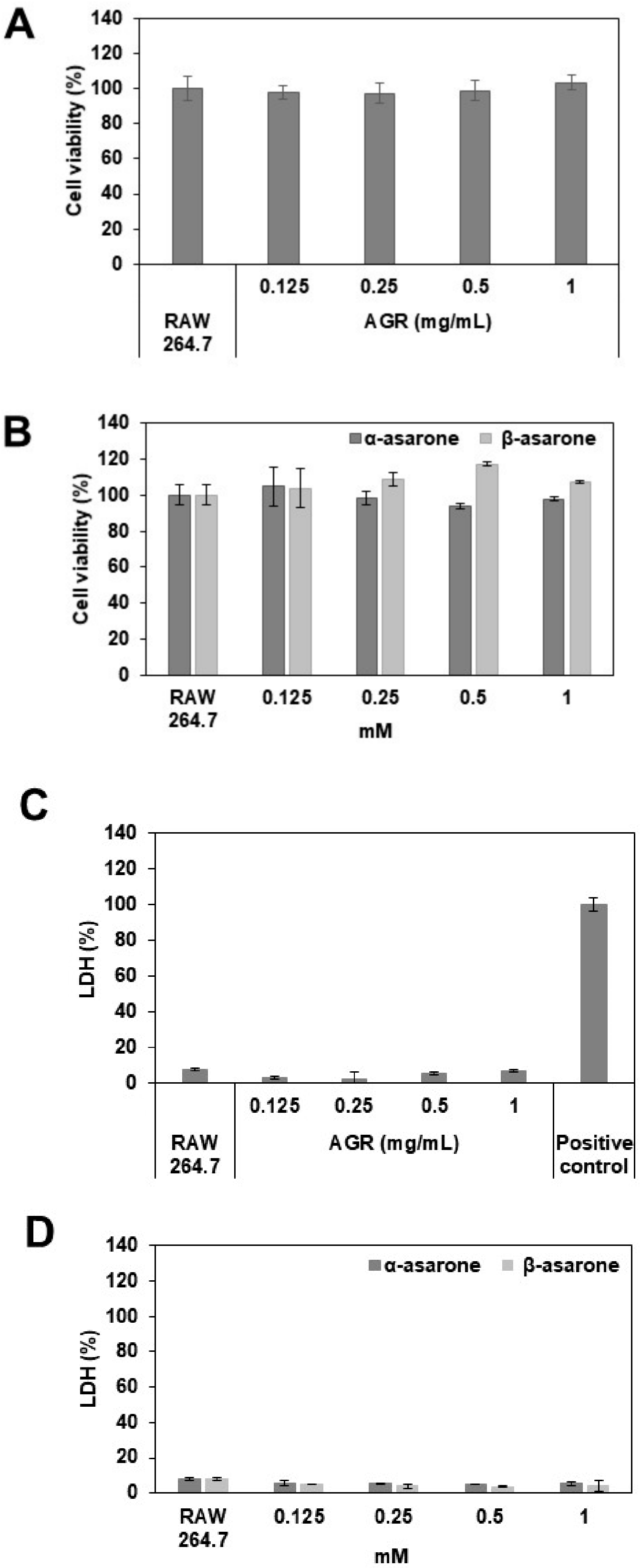

Figure S2. Cytotoxicity of AGR extract, α-asarone, and β-asarone using (A, B) the MTT and (C, D) LDH assays. RAW cells were incubated with the AGR, α-asarone, β-asarone, or combination of α- and β-asarone for 24 h, respectively. The mean value of LDH of the positive control (lysis solution; 0.1% triton X-100) was considered as 100%.
